# A Case Report on Miliary Tuberculosis in Acute Immune Reconstitution Inflammatory Syndrome

**DOI:** 10.21980/J81H02

**Published:** 2020-07-15

**Authors:** Erica Concors, Hamid Ehsani-Nia, Michael Mirza

**Affiliations:** * Rutgers Robert Wood Johnson Medical School, Department of Emergency Medicine, New Brunswick, NJ

## Abstract

This is a case report of an HIV-positive patient with miliary tuberculosis (MTB). He presents to the emergency department (ED) two-weeks following the initiation of antiretroviral therapy (ART) with worsening cough, fevers, and malaise. The patient was diagnosed with Tuberculosis-Immune Reconstitution Inflammatory Syndrome (TB-IRIS) and subsequently treated with corticosteroids, resulting in clinical improvement over a two-week in-patient hospitalization. This case report details a rare case of TB-IRIS, a clinical diagnosis without universally agreed upon criteria. It also demonstrates a classic miliary pattern of disseminated tuberculosis on chest radiography.

**Topics:**

Infectious disease, respiratory, tuberculosis, milary tuberculosis.

**Figure f1-jetem-5-3-v25:**
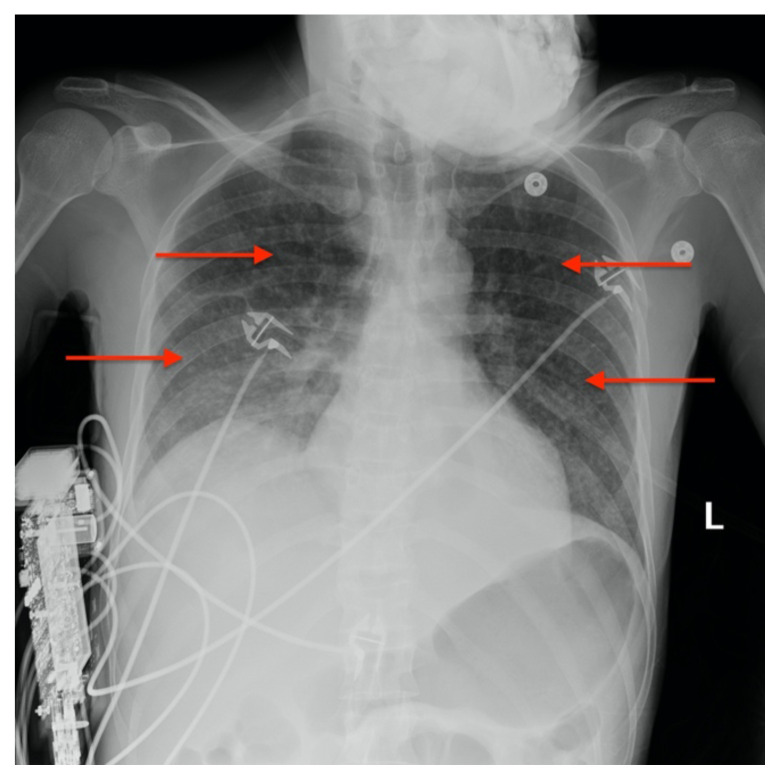


**Figure f2-jetem-5-3-v25:**
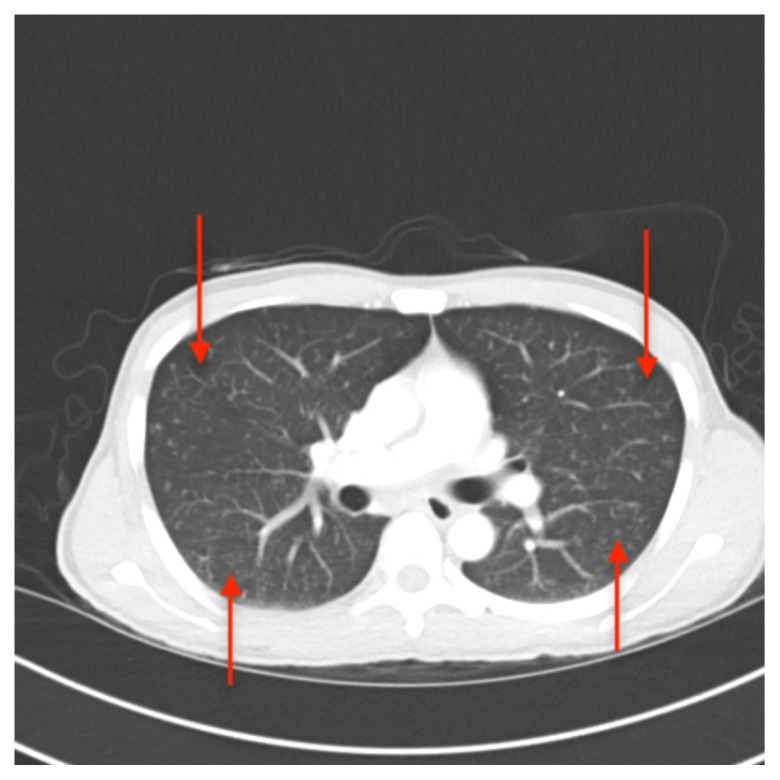


## Introduction

[Fig f1-jetem-5-3-v25][Fig f2-jetem-5-3-v25]Approximately one million people living with HIV worldwide develop Tuberculosis (TB).^1^ Miliary Tuberculosis is the severe manifestation of systemically disseminated Mycobacterium tuberculosis which occurs if a host’s immune system is unable to contain a recently acquired or dormant infection and is common among patients with HIV.^2^

Miliary TB accounts for approximately one percent of all TB cases worldwide.^3^ One potential complication of miliary TB among HIV-positive patients is the immune reconstitution inflammatory syndrome (IRIS). IRIS typically results in the first few months of antiretroviral treatment (ART) and is the result of restoring immune responses to pathogens, commonly from opportunistic infections such as mycobacteria, herpesviruses, or deep fungal infections.^4^

TB-IRIS typically presents in one of two ways: in patients on TB treatment after they initiate HAART (paradoxical TB-IRIS), or in patients diagnosed with TB after starting ART (unmasking TB-IRIS). In paradoxical TB-IRIS, patients develop “recurrent, new, or worsening symptoms or signs of tuberculosis...or deteriorating radiological manifestations,” within the first weeks to three months of initiation of ART therapy.^4^ In this report we examine a case of a patient with paradoxical TB-IRIS. In countries with a low burden of disease, it is important to understand this potentially deadly complication of tuberculosis among at-risk populations, such as patients with extrapulmonary tuberculosis, in order to promptly initiate treatment and reduce overall morbidity and mortality.^5^

## Presenting concerns and clinical findings

A 47-year-old male with history of extrapulmonary tuberculosis on rifampin, isoniazid, pyrazinamide, and ethambutol (RIPE) therapy and recent diagnosis Human immunodeficiency virus type 1 (HIV) presented to the emergency department (ED) complaining of fevers and generalized weakness. The patient was diagnosed with disseminated Tuberculosis and HIV approximately one month prior to presentation, when he was started on RIPE therapy. The administration of highly active antiretroviral therapy (HAART) was intentionally not administered at that time in hopes of avoiding IRIS. The patient was placed on Triumeq® (Abacavir + Dolutegravir + Lamivudine) by his outpatient provider approximately two weeks prior to ED presentation. At that time, the patient’s viral load was 1.7 million copies/mL and his CD4 count was 28 cells/mm^3^.

Approximately two days prior to presentation, the patient began to develop intermittent fevers (T-max 102°F), chills, body aches, a dry cough, and nasal congestion. The patient was not taking his sodium tabs as instructed (taking once daily instead of every eight hours) but otherwise reported compliance with his medications including HAART, Sulfamethoxazole/Trimethoprim prophylaxis, and RIPE. Patient’s vital signs on initial evaluation in the ED showed a temperature of 100.9°F, a heart rate of 136, blood pressure of 114/65, respiratory rate of 18, oxygen saturation of 98% on room air. The initial physical exam was notable for cachexia, dry mucous membranes, and supraclavicular lymphadenopathy. Labs showed a hemoglobin of 9.0g/dL, white blood cell count of 5.9 × 103 cells/mm3, INR: 1.46. Na: 125 mmol/L, albumin 2.6 g/dL, Alkaline phosphatase: 121 IU/L, AST: 77 IU/L, and ALT: 41 IU/L.

## Patient Course

In the ED, the patient received broad spectrum antibiotics (vancomycin 20 mg/kg IV, piperacillin-tazobactam 4.5 gm IV, azithromycin 500 mg IV) and a 1.5 L (30 ml/kg) normal saline IV bolus due to concerns for sepsis. The patient’s vital signs normalized in the ED, and he was admitted. Follow up labs over the next few days showed an improvement in the patient’s CD4 count from 28 cells/mm^3^ to 219 cells/mm^3^. Viral load decreased from 1.7 million copies/mL of HIV to 1,520 copies/mL. An extensive infectious disease work-up including blood and urine cultures, respiratory viral panel, urine legionella antigen was obtained, all of which returned negative. CT of the chest confirmed diffuse pulmonary micronodules and tree-in-bud nodularity suggestive of miliary tuberculosis.

The patient was diagnosed with paradoxical tuberculosis-immune reconstitution inflammatory syndrome (paradoxical TB-IRIS), given the absence of other infectious etiologies and in the setting of a positive response to concomitant HAART and RIPE therapy. An infectious disease consult was obtained who recommended continuation of HAART, Sulfamethoxazole/Trimethoprim prophylaxis, RIPE therapy and initiation of oral corticosteroids. The patient’s hospital course was complicated by recurrent fevers, sinus tachycardia, a right upper lobe pulmonary embolism, persistently up trending transaminitis, and persistent hyponatremia.

He was discharged on day five of 14 of his prednisone course with a plan to taper over a two-week period. His right upper lobe pulmonary embolism was considered provoked secondary to paradoxical TB-IRIS. He was started on apixaban and was on day five of seven of the loading dose at the time of discharge with plans to transition to 5mg twice a day following completion of the loading dose. His persistent hyponatremia improved with some response to mild fluid restriction and initiation of oral salt tabs. His persistently up trending transaminitis was attributed to HAART and/or RIPE therapy and was deferred to the outpatient setting. At the time of discharge, the patient’s overall clinical state was much improved. He was instructed to follow-up with his HIV specialist.

## Significant findings

A portable single-view radiograph of the chest was obtained upon the patient’s arrival to the ED resuscitation bay that showed diffuse reticulonodular airspace opacities (red arrows) seen throughout the bilateral lungs, concerning for disseminated pulmonary tuberculosis. Subsequently, a computed tomography (CT) angiography of the chest was obtained which again demonstrates this diffuse reticulonodular airspace opacity pattern (red arrows).

## Discussion

There is a broad differential diagnosis of the miliary pattern on chest radiography that includes miliary tuberculosis, histoplasmosis, sarcoidosis, pneumoconiosis, bronchoalveolar carcinoma, pulmonary siderosis, and hematogenous metastases.^6^,^7^ The diagnostic accuracy of chest radiographs in identifying patients with miliary tuberculosis is fairly high, with a sensitivity of 59% to 69% and specificity of 97% to 100% with good interobserver agreement (90%).^8^ In this patient, chest radiography demonstrated a miliary pattern common in disseminated TB.

The healthcare burden of TB-IRIS is large, with one 2007 study showing nearly half of patients with TB-IRIS events required hospitalizations for a median of seven days.^9^ The clinical severity of TB-IRIS ranges from mild impairment to respiratory failure and death, and symptoms should only be attributed to the disease after ruling out medication non-compliance or inadequate anti-mycobacterial coverage.^10^ Treatment for TB-IRIS includes continuation of both ART and anti-tuberculosis therapies, as well as anti-inflammatory agents.^11^ With the timely involvement of the Infectious Disease consulting service, this patient was placed on corticosteroids for treatment of TB-IRIS with clinical improvement over a two-week hospital course.

According to a 2011 randomized-control trial (RCT), a four-week course of prednisone reduced inpatient days, outpatient therapeutic procedures, and resulted in more rapid improvements in symptoms, radiography, markers of inflammation, performance, and quality of life.^12^

Current United State Department of Health and Human Services guidelines recommend early ART initiation in patients with TB and severe CD4 T-Cell depletion.^13^ In light of these recommendations, TB-IRIS is an important condition for ED physicians to understand and identify. Biomarkers are a potentially useful tool for ED physicians to improve their evaluation of patients with suspected immune reconstitution inflammatory syndrome. For example, one 2010 cohort study of HIV-positive patients demonstrated a significant increase in plasma levels of D-dimer that approximated the onset of unmasking IRIS. 14 More research is needed, however, to clarify the impact of these biomarkers on overall patient morbidity and mortality.

## Supplementary Information








