# Supplementary figures and images for: A Case Report on Miliary Tuberculosis in Acute Immune Reconstitution Inflammatory Syndrome
Source: J Educ Teach Emerg Med. 2020 Jul 15;5(3):V25–8. doi: 10.21980/J81H02 (PMC10332554; doi:10.21980/J81H02)

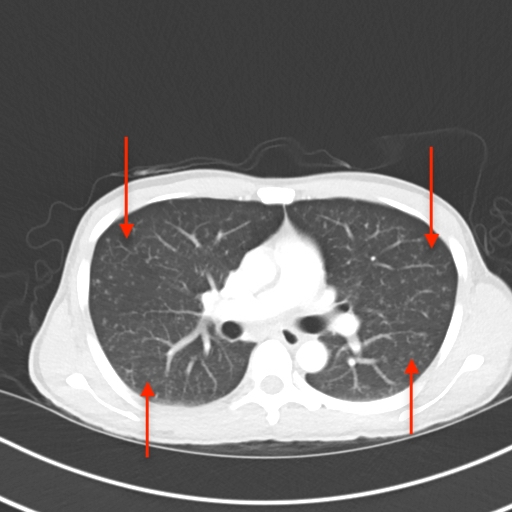

Supplement: Supplementary file 1 [file jetem-5-3-v25-supp1.jpg]

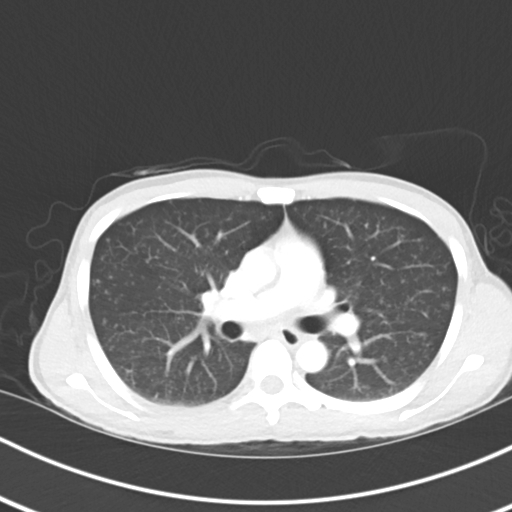

Supplement: Supplementary file 2 [file jetem-5-3-v25-supp2.jpg]

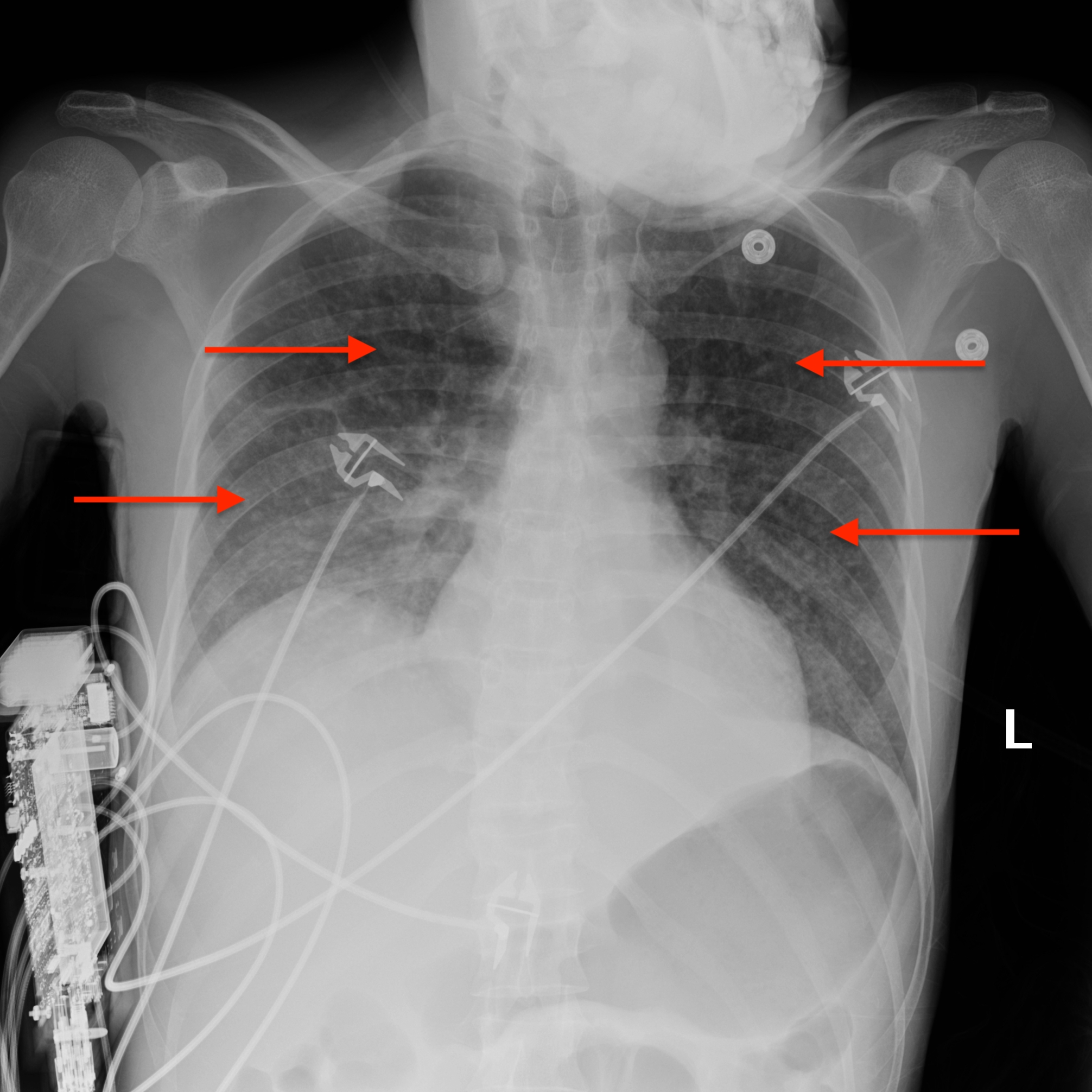

Supplement: Supplementary file 3 [file jetem-5-3-v25-supp3.jpg]

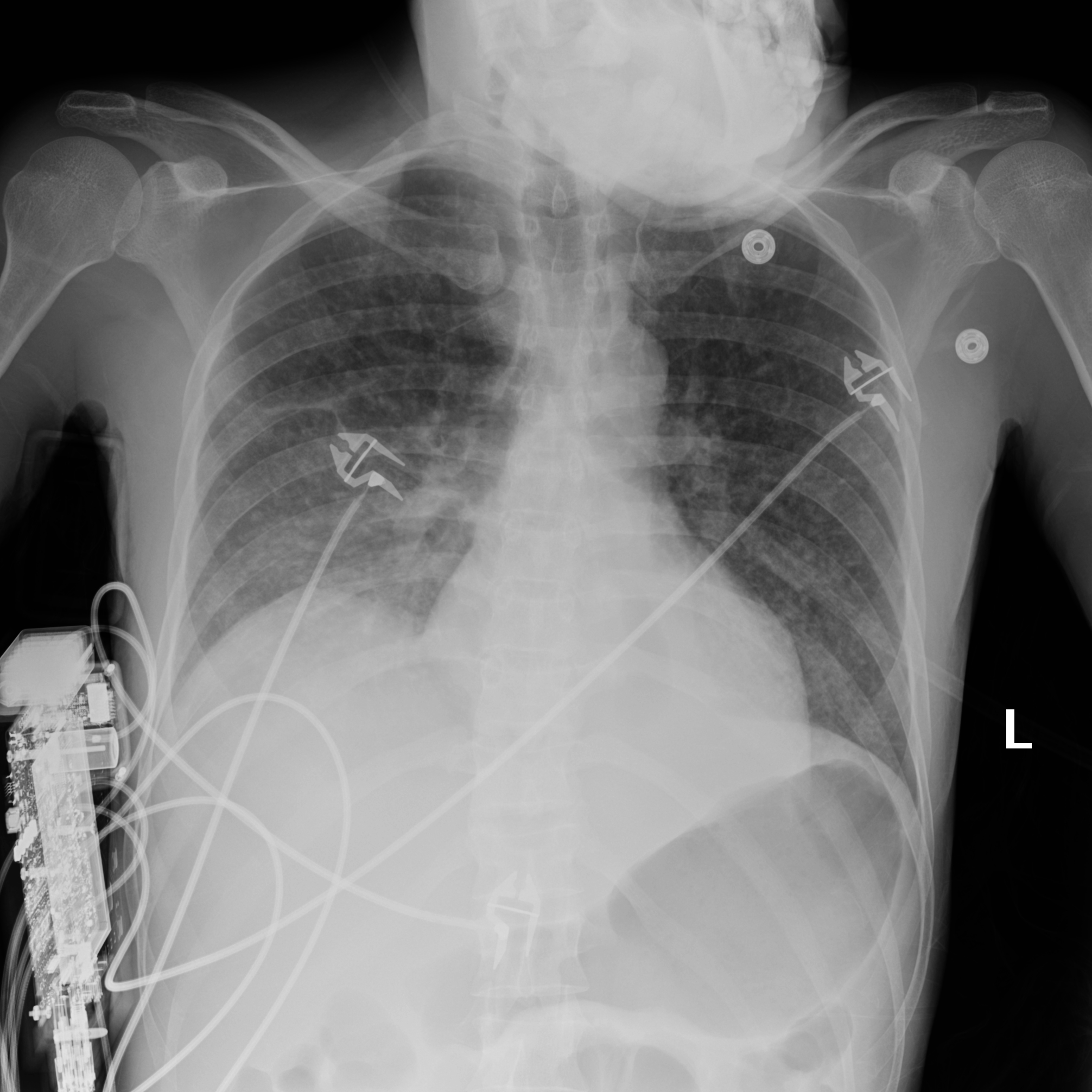

Supplement: Supplementary file 4 [file jetem-5-3-v25-supp4.jpg]
